# Delta-radiomics features of ADC maps as early predictors of treatment response in lung cancer

**DOI:** 10.1186/s13244-024-01787-5

**Published:** 2024-08-26

**Authors:** Christian M. Heidt, Jonas R. Bohn, Róbert Stollmayer, Oyunbileg von Stackelberg, Stephan Rheinheimer, Farastuk Bozorgmehr, Karsten Senghas, Kai Schlamp, Oliver Weinheimer, Frederik L. Giesel, Hans-Ulrich Kauczor, Claus Peter Heußel, Gudula Heußel

**Affiliations:** 1 Diagnostic and Interventional Radiology, Heidelberg University Hospital, Heidelberg, Germany; 2 Translational Lung Research Center (TLRC), Member of the German Center for Lung Research (DZL), Heidelberg, Germany; 3 Division of Medical Image Computing, German Cancer Research Center, Heidelberg, Germany; 4 Faculty of Biosciences, Heidelberg University, Heidelberg, Germany; 5 National Center for Tumor Diseases (NCT Heidelberg), Heidelberg, Germany; 6 Diagnostic and Interventional Radiology with Nuclear Medicine, Thoraxklinik at University Hospital Heidelberg, Heidelberg, Germany; 7 Thoracic Oncology, Thoraxklinik at University Hospital Heidelberg, Heidelberg, Germany; 8 Section for Translational Research, Thoraxklinik at University Hospital Heidelberg, Heidelberg, Germany; 9 Department of Nuclear Medicine, Medical Faculty, Heinrich-Heine-University, University Hospital Düsseldorf, Düsseldorf, Germany; 10 Pneumology and Respiratory Critical Care Medicine, Thoraxklinik at University Hospital Heidelberg, Heidelberg, Germany

**Keywords:** Radiomics, Lung cancer, Diffusion-weighted MRI, Non-small cell lung cancer, Tyrosine kinase inhibitors

## Abstract

**Objective:**

Investigate the feasibility of detecting early treatment-induced tumor tissue changes in patients with advanced lung adenocarcinoma using diffusion-weighted MRI-derived radiomics features.

**Methods:**

This prospective observational study included 144 patients receiving either tyrosine kinase inhibitors (TKI, *n* = 64) or platinum-based chemotherapy (PBC, *n* = 80) for the treatment of pulmonary adenocarcinoma. Patients underwent diffusion-weighted MRI the day prior to therapy (baseline, all patients), as well as either + 1 (PBC) or + 7 and + 14 (TKI) days after treatment initiation. One hundred ninety-seven radiomics features were extracted from manually delineated tumor volumes. Feature changes over time were analyzed for correlation with treatment response (TR) according to CT-derived RECIST after 2 months and progression-free survival (PFS).

**Results:**

Out of 14 selected delta-radiomics features, 6 showed significant correlations with PFS or TR. Most significant correlations were found after 14 days. Features quantifying ROI heterogeneity, such as short-run emphasis (*p* = 0.04_(pfs)_/0.005_(tr)_), gradient short-run emphasis (*p* = 0.06_(pfs)_/0.01_(tr)_), and zone percentage (*p* = 0.02_(pfs)_/0.01_(tr)_) increased in patients with overall better TR whereas patients with worse overall response showed an increase in features quantifying ROI homogeneity, such as normalized inverse difference (*p* = 0.01_(pfs)_/0.04_(tr)_). Clustering of these features allows stratification of patients into groups of longer and shorter survival.

**Conclusion:**

Two weeks after initiation of treatment, diffusion MRI of lung adenocarcinoma reveals quantifiable tissue-level insights that correlate well with future treatment (non-)response. Diffusion MRI-derived radiomics thus shows promise as an early, radiation-free decision-support to predict efficacy and potentially alter the treatment course early.

**Critical relevance statement:**

Delta-Radiomics texture features derived from diffusion-weighted MRI of lung adenocarcinoma, acquired as early as 2 weeks after initiation of treatment, are significantly correlated with RECIST TR and PFS as obtained through later morphological imaging.

**Key Points:**

- Morphological imaging takes time to detect TR in lung cancer, diffusion-weighted MRI might identify response earlier.
- Several radiomics features are significantly correlated with TR and PFS.
- Radiomics of diffusion-weighted MRI may facilitate patient stratification and management.

**Graphical Abstract:**

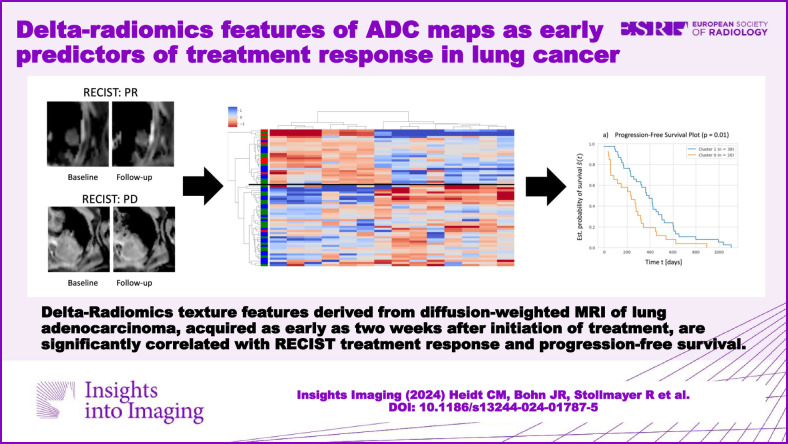

## Introduction

Lung cancer ranks third in cancer diagnoses for both sexes, following prostate and breast cancer [1]. It is also the most common cause of cancer-related death worldwide. Early-stage lung cancers often remain asymptomatic and, by the time symptomatic cases are diagnosed, the disease has often progressed to a locoregionally advanced stage, resulting in poor prognosis and limited treatment options [2].

Non-small cell lung cancer (NSCLC) constitutes the majority of all lung cancer diagnoses [2]. Once NSCLC advances beyond surgical resectability, treatment options include chemotherapy, radiotherapy, as well as targeted treatments with tyrosine kinase inhibitors (TKI) or immune checkpoint inhibitors [3]. Each of these treatment options carries the risk of significant side effects for the patient [4], it is therefore important to select the regimen with the highest chance of success while monitoring patients for treatment response (TR).

Currently, TR is usually assessed in intervals of several months on follow-up CT imaging according to response evaluation criteria in solid tumors (RECIST), a guideline that stems from visual inspection of target lesions [5]. Especially in the lung, this method faces several challenges: it is impeded by co-findings such as retention pneumonia, atelectasis, or pleural effusions hiding the underlying tumor mass [6]. Focusing only on tumor axis changes discounts other factors that might indicate TR. Additionally, the long intervals of the CT follow-up schedule are not conducive to assessing a possible TR early on in a treatment regimen. Earlier detection of non-responders would allow termination of ineffective treatment courses ahead of time, reducing the burden on patients and potentially allowing adjustment to a different treatment plan [7]. On the other hand, symptomatic treatment of potential side effects may be strengthened upon prediction of TR.

Radiomics is an approach that quantifies imaging data into features that can then be analyzed for clinical correlations [8, 9]. Recently, imaging biomarker analysis with radiomics has been proposed as an automated way of analyzing tissue changes beyond the morphology [10]. Assessing the development of these imaging features throughout a treatment regimen is known as delta-radiomics analysis and has been shown to be predictive of TR in several studies [11, 12]. Although frequently used in chest CT imaging, delta-radiomics applied to functional imaging remains understudied. Previously, functional imaging, such as perfusion-MR [13] or diffusion-weighted MR imaging (DWI), has shown earlier detection of tissue changes compared to morphological imaging [14].

The purpose of this study is to analyze the feasibility of extracting radiomic features from DWI of the lung early on in the course of a treatment regimen and using Delta-Radiomics analysis to evaluate their bearing on TR.

## Materials and methods

### Patient data

For this prospective observational early response trial, a cohort of 144 patients with primary adenocarcinoma of the lung were recruited. The median patient age was 64 years (range 41–86 years). Treatment was administered according to guidelines, therefore patients with a confirmed epidermal growth factor receptor mutation (*n* = 64) received treatment with TKIs, while EGFR-negative patients (*n* = 80) were treated with platinum-based chemotherapy (PBC). Due to a change in treatment guidelines, a small number (*n* = 17) of patients undergoing PBC also received Pembrolizumab. Patients with metal implants close to the tumor were excluded. This study was performed in accordance with the Declaration of Helsinki and was approved by the Ethics Committee of the Heidelberg Medical Faculty—approval: S-445/2015. All adult participants provided written informed consent to participate in this study.

### Image acquisition protocol

For each patient, both a chest CT, as well as a diffusion-weighted MRI with the generation of apparent diffusion coefficient (ADC) maps of the primary tumor was performed at baseline (BL) (day 0) before treatment initiation. Follow-up diffusion-weighted MRI was performed in the TKI cohort on days 7 (follow-up 1 (FU1)) and 14 (follow-up 2 (FU2)) after treatment initiation and in the PBC cohort on day 1 due to regulations in the German DRG system. Patients undergoing PBC with Pembrolizumab were available for a second DWI follow-up after 7 days. Each MRI appointment further included the acquisition of T1 VIBE and T2 HASTE sequences, however, the focus of this study rests on assessing the early changes in functional DWI.

TR was assessed based on CT imaging according to RECIST 1.1 [5], with classifications of complete response (CR), partial response (PR), stable disease (SD), and progressive disease (PD). The first respective follow-up CTs were performed 2 months after initiation of treatment and were used as TR for this study. Further follow-up CTs were performed in 3–6-month intervals.

All diffusion imaging was performed on a Siemens Aera 1.5-T scanner using a 2D diffusion sequence, the details of which can be found in Table 1. CT imaging was performed on a Siemens Somatom Definition AS scanner.

**Table 1:** Protocol used for the DWI acquisition

Device	Siemens Aera 1.5 T
Sequence	EPI
Columns × rows [pixel]	104 × 136
FOV [mm]	344 × 450
Parallelization factor	2
Slices	25
Slice thickness [mm]	5
Spacing [mm]	6
TR [ms]	3800
TE [ms]	65
Averages	4
Flip angle [°]	90
Bandwidth [Hz/pixel]	1225
*B*-values [s/mm^2^]	0, 50, 150, 500, 1000
Total acquisition time [min]	3:37 min

*FOV* field of view, *TR* repetition time, *TE* echo time

The primary tumor regions of interest (ROIs) for extraction of radiomic features from ADC maps were manually segmented on the ADC map by a thoracic radiologist with 29 years of experience while simultaneously consulting the CT and B_1000_ DWI sequence. Segmentation was carried out using MINT Lesion 3.7.3, excluding extratumoral signal sources such as pleural effusions or atelectasis. RECIST measurements on CT imaging were also carried out by an expert thoracic radiologist using MINT Lesion 3.7.3.

### Feature extraction

Feature extraction has been performed using the PyRadiomics library v3.0.1 [15]. Before feature extraction, the ADC maps were discretized using a fixed bin size of 50. The following features were extracted from the ROIs, parentheses indicating their image biomarker standardization initiative (IBSI)-defined abbreviations: shape (MORPH), first-order features (IS, IH, and IVH), gray-level co-occurrence matrix (GLCM), gray-level run-length matrix (GLRLM), gray-level size-zone matrix (GLSZM), gray-level distance-zone matrix, neighborhood gray-level difference matrix [16]. These features were extracted both from the original images, as well as the gradient magnitude images, for a total of 197 features.

For analysis of the feature implications at given time points, the features of that time point were used. For longitudinal analysis of the implications of delta-radiomics features (DRFs), the DRFs were calculated as follows, where *t*_*i*_ denotes time point *i* and *f* denotes the value of a given feature at that time point: $\Delta {f}_{{t}_{i}}=\frac{{f}_{{t}_{i}}-{f}_{{t}_{0}}}{{f}_{{t}_{0}}}$. Given the highly different numerical ranges of different radiomic features, features were normalized using *Z*-score normalization. To reduce the number of features before analysis, feature selection has been performed with progression-free survival (PFS) as the target variable. Features with a Pearson correlation of *r*_p_ < 0.2 were eliminated. In the resulting feature sets, multicollinearity between features has been assessed and features with a correlation coefficient *r*_p_ > 0.85 have been removed.

### Statistics

Exploratory clustering, to identify similarly expressed clusters of patients in the cohort, has been performed using Ward’s minimum variance method.

Unless otherwise stated, the significance of the resulting features with regard to the target variable has been assessed by performing *t*-tests, with a *p*-value of < 0.05 being considered significant. Correction for multiple testing was performed according to Holm’s method.

Survival estimates have been calculated using Kaplan–Meier estimators and evaluated using log-rank tests.

## Results

**Fig. 1:**
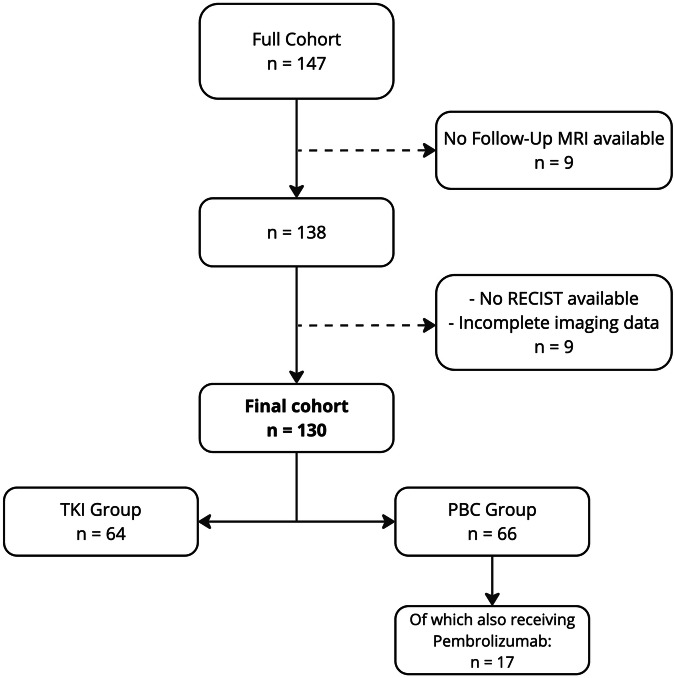
Flowchart of the study. TKI, tyrosine-kinase inhibitors; PBC, platinum-based chemotherapy

In total, 130 patients were included in the study (Fig. 1). PFS was recorded for 124 patients. After treatment initiation, the median PFS was 231 days (interquartile range (IQR): 121–419 days). The overall survival (OS) was recorded for 91 patients, with a median OS of 309 days (IQR: 162–579 days). Response classification using RECIST on the first follow-up CT at 8 weeks yielded 40 patients with PR, 80 patients with SD, and 10 patients with PD. Specifics for the two treatment subgroups can be found in Table 2. Of the PBC group, only those patients receiving PBC together with Pembrolizumab were available for a second follow-up MRI after 7 days.

**Table 2:** Subgroup-specific information about the study cohort

	TKI			PBC		
Day	0	∆7 d	∆14 d	0	∆1 d	∆7 d
Amount [*n*]	64	64	60	66	66	17
Median age [yrs] (range)		65 (41–85)			64 (45–86)	
Sex (f | m)		42 | 22			29 | 37	
RECIST: PR		32			8	
RECIST: SD		27			53	
RECIST: PD		5			5	
Median PFS [d] (IQR)		363 (157–507)			180 (94–294)	
Median OS [d] (IQR)		441 (189–721)			281 (161–460)	

RECIST response classification was carried out on the first follow-up CT after 8 weeks

*TKI* tyrosine-kinase inhibitors, *PBC* platinum-based chemotherapy

### Selected DRFs

After eliminating features with a low correlation to PFS, as well as features containing redundant information, a set of 14 DRFs was obtained. The selected features are specified in Table 3. This feature set was evaluated across all time points and patient groups to ensure consistency.

**Table 3:** Overview of the final set of DRFs considered in the evaluation

Image aspect	Feature family	Feature (IBSI)
Original	Shape	SurfaceVolumeRatio (*2PR5*)
		MinorAxisLength (*P9VJ*)
	Intensity	Kurtosis (*IPH6*)
	GLSZM	ZonePercentage (*P30P*)
		ZoneEntropy (*GU8N*)
		SmallAreaLowGrayLevelEmphasis (*5RAI*)
	GLRLM	ShortRunEmphasis (*22OV*)
	GLCM	InverseVariance (*E8JP*)
		InformationalMeasureOfCorrelation1 (*R8DG*)
		InverseDifferenceNormalized (IDN) (*NDRX*)
Gradient	Intensity	Kurtosis (*IPH6*)
	GRLRM	ShortRunEmphasis (*22OV*)
		RunEntropy (*HJ9O*)
	GLCM	InverseDifferenceNormalized (*NDRX*)

Enclosed in parentheses are the unique permanent identifiers for each feature as proposed by the IBSI

### Clustering and survival analysis

**Fig. 2:**
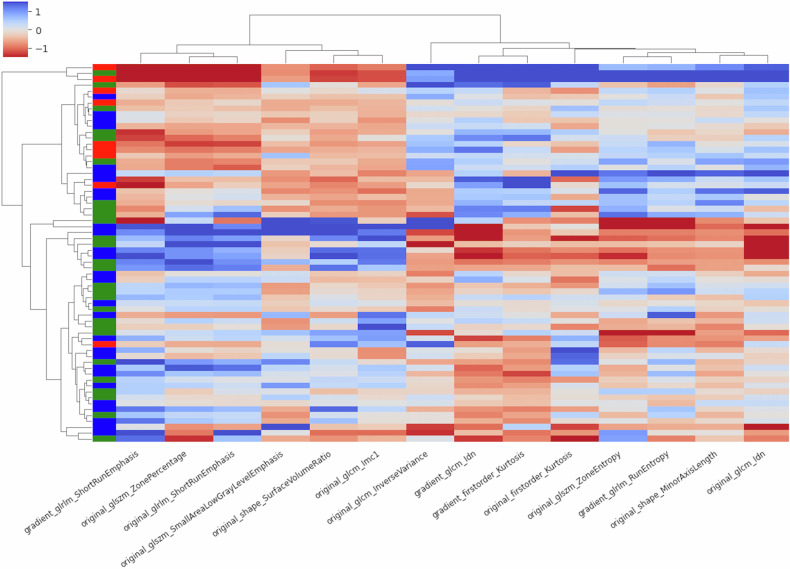
Hierarchical clustering heatmap of changes in feature expression at FU2. Rows signify patients, columns signify features. The color coding next to the heatmap indicates RECIST classification at the first follow-up CT (red: PD, blue: SD, and green: PR). Well visible are two distinct groups of patients, one with decreased expression of the first 6 features and an increase of the remaining, the second with the reversed pattern. The horizontal black bar defines the separation between cluster 1 (top) and cluster 2 (bottom)

Exploratory clustering of the normalized DRFs showed a visible distinction between patient groups. Figure 2 shows a clustered heatmap of DRF expression, with each row of the heatmap corresponding to one patient and each column to one feature. We found two distinct clusters. Cluster 1 consisted of 40% of patients with decreased expression in the first six features (left to right), while the remaining features had an increase in expression compared to BL. Cluster 2 exhibited a reversed pattern with 60% of the patients showing an increase in the first 6 features and a decrease in the remaining.

The color coding next to the heatmap on the left encodes the RECIST evaluation for each patient at the first follow-up CT after 6 weeks, with green bars denoting PR, blue bars denoting SD, and red bars denoting PD. Visibly, cluster 1 contains a lower concentration of cases with PR and contains 9 out of 10 cases with confirmed PD.

**Fig. 3:**
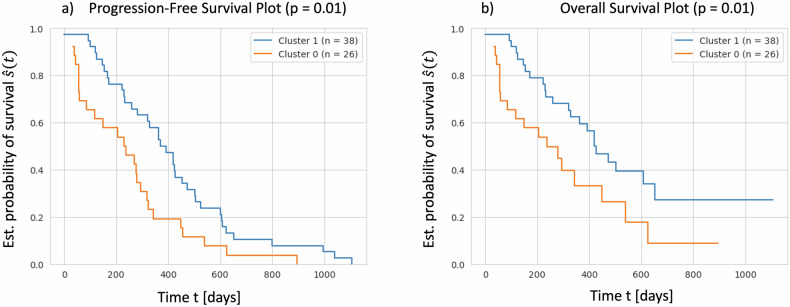
Kaplan–Meier estimators illustrating (**a**) PFS functions, (**b**) OS functions between the identified patient clusters from Fig. 2. Cluster 2 shows prolonged survival for both metrics as opposed to cluster 1, with PFS being significantly prolonged at *p* = 0.01 and OS showing a similar tendency at *p* = 0.06 after applying log-rank test. The OS function contains right-censored data of 22 patients lost to follow-up

These differentially expressed clusters of DRFs after 14 days of treatment could be shown to correlate to clinical outcomes. We performed a Kaplan–Meier estimator of PFS and OS for both clusters. We found cluster 2 to have a significantly longer PFS (*p* = 0.01) compared to cluster 1 (Fig. 3a) and a tendency (*p* = 0.06) to be prolonged in OS (Fig. 3b).

### Feature analysis

The change in feature expression has been evaluated for both treatment subgroups and for both follow-up DWI time points. Regarding PFS, the data for the *t*-tests was separated into two groups: patients with a PFS > 365 days and patients with a PFS ≤ 365 days. Regarding TR, the data was separated into two groups: patients with PD according to the RECIST evaluation of the first follow-up CT at 8 weeks and patients with SD or PR.

Based on the *t*-test results, 6 out of the 14 selected DRFs were significantly correlated to either PFS or TR (Table 4). Most significant correlations for both outcomes were found for the FU2 difference in feature expressions compared to BL. Earlier time points, namely evaluations after one day for patients receiving PBC or after seven days for patients receiving TKI, showed fewer significantly correlated features. No statistical differences were found between PR and SD expressions for any timeframe or treatment group.

**Table 4:** Mean feature expression change and corresponding *p*-values of all features significantly correlated with either TR or PFS

	FU1						FU2								
	TR														
	PBC			TKI			PBC			TKI			All		
Feature	µ (PR and SD)	µ (PD)	*p*	µ (PR and SD)	µ (PD)	*p*	µ (PR and SD)	µ (PD)	*p*	µ (PR and SD)	µ (PD)	*p*	µ (PR and SD)	µ (PD)	*p*
ZP	0.04	− 0.03	0.99	0.18	− 0.06	0.71	0.01	− 0.20	0.90	**0.25**	− **0.10**	**0.04**	**0.20**	− **0.14**	**0.01**
SRE	0.00	0.00	0.99	0.01	− 0.01	0.62	0.00	− 0.01	0.93	**0.01**	− **0.01**	**0.01**	**0.01**	− **0.01**	**0.005**
Kurtosis	0.10	0.02	0.99	− **0.06**	**0.56**	**0.05**	0.09	0.40	0.96	− 0.12	0.08	0.65	− 0.08	0.19	0.45
G_SRE	0.01	− 0.02	0.99	0.08	0.00	0.82	0.03	− 0.11	0.86	0.08	− 0.02	0.10	**0.07**	− **0.05**	**0.01**
G_IDN	0.00	0.00	0.99	− 0.03	0.01	0.82	0.01	0.05	0.92	− 0.03	0.01	0.19	− **0.03**	**0.02**	**0.04**

	PFS														
	PBC			TKI			PBC			TKI			All		
Feature	µ (> 1 y)	(µ ≤ 1 y)	*p*	µ (> 1 y)	µ (≤ 1 y)	*p*	µ (> 1 y)	µ (≤ 1 y)	*p*	µ (> 1 y)	µ (≤ 1 y)	*p*	µ (> 1 y)	µ (≤ 1 y)	*p*
ZP	− 0.23	0.07	0.10	0.17	0.13	1.00	0.13	− 0.05	0.99	0.30	0.12	0.21	**0.29**	**0.06**	**0.02**
SRE	− **0.02**	**0.00**	**0.03**	0.01	0.01	0.98	0.00	0.00	0.99	0.02	0.00	0.10	**0.02**	**0.00**	**0.04**
Inverse difference normalized	0.02	0.00	0.81	− 0.02	0.00	0.90	− 0.02	0.02	0.99	− 0.03	− 0.01	0.09	− **0.03**	**0.00**	**0.01**
G_IDN	0.02	0.00	0.82	− 0.03	− 0.02	0.99	− 0.02	0.02	0.99	− 0.04	− 0.02	0.19	− **0.04**	**0.00**	**0.01**

Bold font indicates statistically significant results (*p* < 0.05)

**Fig. 4:**
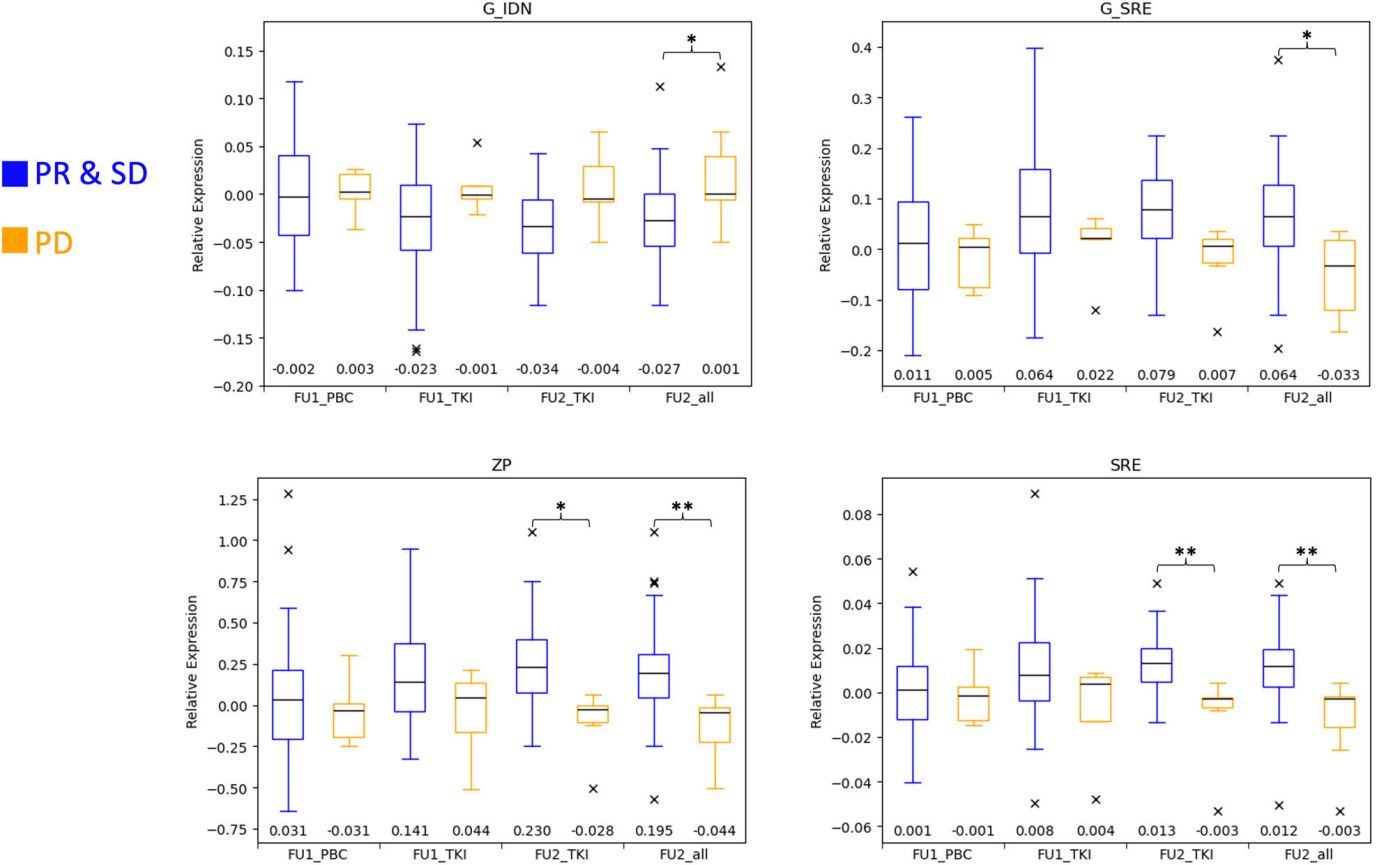
Expression changes between BL and follow-up imaging per group. G_IDN, gradient inverse difference normalized; SRE, short-run emphasis; G_SRE, gradient SRE; ZP, zone percentage; FU1, follow-up 1; FU2, follow-up 2. *p < 0.05, **p < 0.01

A comparison of differential DRF expression between groups can be found in Fig. 4, depicting the differential expression of four DRFs at different follow-ups. It can be seen that in the group with PR and SD features such as *SRE* and *ZP* show an increase when compared to their expression at treatment initiation. Simultaneously, other features such as *G_IDN* are decreased compared to the BL.

**Fig. 5:**
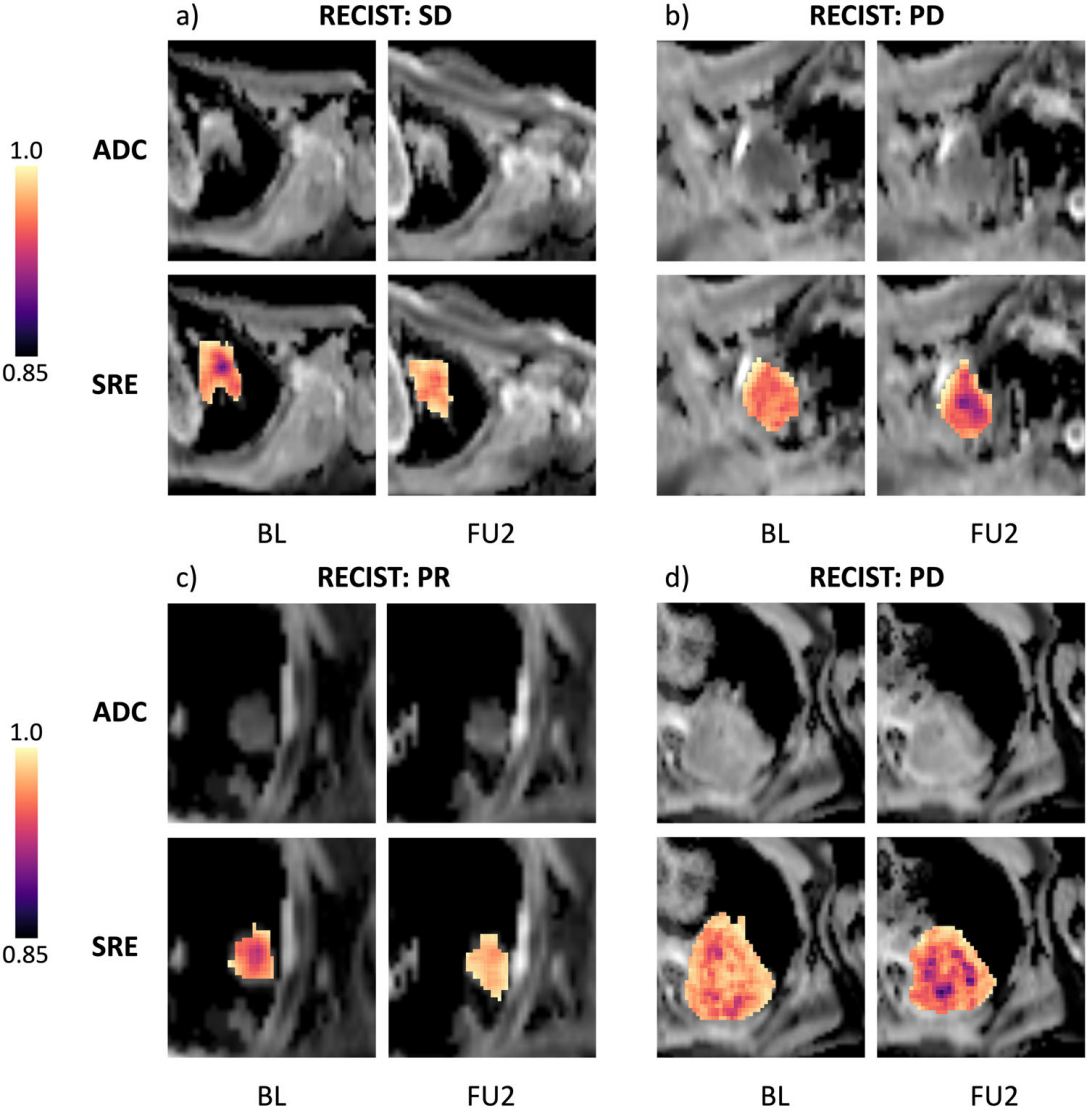
ADC maps and feature maps showing differential expression of feature “ShortRunEmphasis” (SRE) on BL and FU2 imaging (brighter colors mean higher expression). **a** Patient with SD, TKI group (progression: 1039 days). Visible is an increase in SRE as a brightening especially in the center of the ROI; a similar effect is visible in (**c**) patient with PR, TKI (progression: 419 days). Opposed are (**b**) patient with PD, TKI group (progression: 56 days) with a visible decrease in SRE and (**d**) patient with PD, PBC group (progression: 40 days)

To visualize these changes, we overlayed patient feature maps and ADC maps (Fig. 5). As a representative example, we displayed the SRE feature as it correlated with both PFS and TR. The overlayed maps depict the apparent rise in PR and SD from BL to FU2 (Fig. 5a, c), and the decrease in PD cases (Fig. 5b, d).

## Discussion

In this study, we investigated the applicability of DWI-derived DRFs for early TR assessment in NSCLC. To the best of our knowledge, this is the first study that investigates DWI-derived DRFs in lung imaging.

Diffusion-weighted imaging is commonly employed in clinical practice as a way to detect functional tissue changes before any morphological changes can be seen in MR or CT imaging [14]. Regarding the applicability of DWI and radiomics to assess TR in NSCLC, our findings partially support this premise. Notably, in DWI performed 14 days after the initiation of TKI treatment, several DRFs, particularly texture features, show a significant correlation with longer PFS. Similar effects have been reported in other tumor types, such as prostate cancer [17]. To better understand the correlation of the selected DRFs to TR or PFS, it is important to consider what these features measure.

### Feature interpretation

When considering the differences in DRF expression, our results showed two patient clusters (Fig. 2). Cluster 2, the cluster with significantly longer PFS, showed an increase in six features, five of which quantify heterogeneity within a ROI (gradient SRE, ZP, SRE, small area low gray level emphasis*,* Imc1*)*. An increase in these features therefore indicates a less homogenous presentation of ADCs in the image, with shorter run lengths and finer textures. The sixth feature quantifies the surface-to-volume ratio, which rises as tumor volume decreases. As tumor volume is typically assessed through CT RECIST examination and DWI is a primarily functional, not morphological, imaging sequence, this feature should be interpreted with caution.

In contrast, the features that are decreased in this cluster quantify homogeneity or tumor growth. For example. a decrease in Kurtosis or *gradient_glcm_Idn* suggests a more heterogenous presentation of ADC values and a decrease in *original_shape_MinorAxisLength* indicates shrinkage of the tumor’s short axis.

The inverse expression of these features within the cluster, the cluster associated with shorter PFS, therefore indicates a link between a stagnant or increasingly homogenous presentation of ADC values within the tumor ROI and less favorable outcomes. These findings are consistent with known properties of tumor tissue in diffusion imaging [18], where increased cellular density restricts diffusion, leading to a darker, more homogeneous presentation of ADC values.

We speculate that in cases with more favorable outcomes, such as a PR or longer PFS, the tumor’s more heterogeneous ADC values reflect a decline in cellular density in response to the treatment. With some cell death resulting from the early stages of treatment, the physical sparsification of cells would explain a patchwise localized increase in diffusivity within previously diffusion-restricted tissue. This effect can be detected through radiomic features such as SRE or ZP. Conversely, stagnant or rising features of homogeneity in DWI can be explained by a high cellular density within the tumor tissue, restricting diffusion uniformly over a larger area. These findings and their correlation with PFS are consistent with histopathological findings of NSCLC, where higher cellular density is negatively correlated with PFS [19].

Uniquely, in the first follow-up of the PBC group, a significant correlation is observed between a decrease in SRE and a PFS greater than 365 days, which contrasts with findings in later follow-ups. This initial decrease may be caused by cell swelling during the uptake of platinum, before cell death occurs, leading to locally more restricted diffusion.

The main finding of this study is thus, that diffusion-weighted imaging of the lung can provide insights into TR in patients with NSCLC as early as 14 days after treatment initiation. Earlier follow-up imaging, such as after 1 (PBC) or 7 (TKI) days, while also showing correlations with PFS or TR, does not yet reliably capture this link, as is visible in the respective columns of Table 4.

This discrepancy can be explained in part by the nature of treatment administration. PBC is administered intravenously and quickly reaches an effective concentration within well-perfused tissues. In contrast, TKIs are administered orally and may take much longer to reach a sufficient concentration to induce cell death. Prior research has observed that orally administered TKIs take effect after about 8 days [20], which may explain the more pronounced and significant change in DRFs at 14-day follow-ups compared to 7-day follow-ups.

### Contextualization with previous work

Prior studies have found increased mean ADC indicative of TR in several tumor entities, such as prostate, breast, and rectal cancer [14, 21]. This increase is typically attributed to tissue sparsification following necrosis and supports our findings of an initial increase in heterogeneity.

In contrast to our findings, a radiomics-based study by Lee et al reports increased mean ADC with a decrease in heterogeneity features after 6 weeks of prostate cancer treatment [22]. It is possible that over this longer follow-up period, necrosis has pervaded the tissue enough to lead to homogenization, whereas, in our short-term observation, necrosis is only occurring locally.

In lung imaging, DWI is not widely applied on a large scale, however, several smaller pilot studies have explored its potential for TR assessment, mostly in radiochemotherapy (RCT). Jagoda et al have found that in DWI follow-up imaging of 12 patients at 3 months, 6 months, and 12 months after the initiation of RCT, patients with PR consistently showed higher mean ADC values than those with PD [23]. In a longitudinal DWI study of 25 patients, Sorgun et al reported that increased ADC values after a full course of RCT had a significant inverse correlation with tumor size change, indicative of response [24]. Similarly, Carlin et al evaluated ADC changes in 14 patients undergoing neoadjuvant chemotherapy and found that responders presented with significantly increased median ADC after 14 days, a timeframe comparable to ours [25].

While DRFs quantifying mean/median ADC change did not meet statistical significance in our observation, we observed a similar tendency as these studies, with higher ADC values in PR and SD cases and lower values in PD cases. This might again be due to differences in follow-up periods, as well as cohort sizes, as significant increases in median ADC following tissue sparsification may take longer to appear than our 14-day follow-up period.

### Limitations

A limitation of this study lies within our follow-up schedule. Due to German DRG regulations, patients receiving PBC had to be followed up within 1 day, and only a small group of these patients could be re-imaged after 7 days. Longer follow-up diffusion imaging could provide clearer insights into future responses to both PBC and TKIs, as suggested by several pilot studies [23, 24].

In addition to the follow-up timeframe, the exact timing of measurements also appears to be an elementary factor to consider in DWI studies. Tumors that respond well to intervention typically show an increase in ADC values, but cases of an initial decrease followed by an increase in ADC values have also been observed [14]. This dynamic is explained by initial cell swelling preceding cell death. Analyzing such changes in NSCLC would potentially benefit from more frequent measurements during the first two weeks than were performed in this study.

The manual segmentation of tumor ROIs does present a potential source of bias, as no inter-reader consensus was established.

Furthermore, it is worth noting that the correlations with TR as reported in Table 4 are based on a comparatively small absolute number of only ten patients with confirmed PD (*n*_(TKI)_ = 5, *n*_(PBC)_ = 5). Although a longer-term correlation with PFS yielded similar results in terms of significantly correlated DRFs for all patients, the study’s overall cohort size remains a limiting factor. For this reason, the analysis is also confined to descriptive statistics. A larger cohort could provide additional insights into the early presentation of various RECIST response groups and allow for predictive modeling.

## Conclusion

This study has investigated the link between changes in radiomic features of ADC maps over time and early TR in patients with NSCLC, aiming to evaluate the viability of DWI for response assessment in lung cancer. We have demonstrated that several DRFs quantifying intratumoral heterogeneity of ADC values and tumor shrinkage significantly correlate with TR and PFS of patients, particularly after 14 days of treatment.

Repeated DWI shows promise for assessing TR and potentially altering treatment courses much earlier than CT imaging. The study suggests further research avenues, such as investigating changes in DWI over a longer time course or the efficacy of DWI-derived DRFs for predictive modeling with machine or deep learning approaches.

## Data Availability

The raw data cannot be made freely available because of privacy restrictions but the datasets used and/or analyzed during the current study are available from the corresponding author on reasonable request.

## Abbreviations

ADC: Apparent diffusion coefficient
BL: Baseline
CR: Complete response
DRFs: Delta-radiomics features
DWI: Diffusion-weighted MR imaging
FU1: Follow-up 1
FU2: Follow-up 2
G_IDN: Gradient inverse difference normalized
G_SRE: Gradient short run emphasis
GLCM: Gray-level co-occurrence matrix
GLRLM: Gray-level run-length matrix
GLSZM: Gray-level size-zone matrix
IBSI: Image biomarker standardization initiative
IQR: Interquartile range
NSCLC: Non-small cell lung cancer
OS: Overall survival
PBC: Platinum-based chemotherapy
PD: Progressive disease
PFS: Progression-free survival
PR: Partial response
RCT: Radiochemotherapy
RECIST: Response evaluation criteria in solid tumors
ROI: Region of interest
SD: Stable disease
SRE: Short run emphasis
TKI: Tyrosine-kinase inhibitors
TR: Treatment response
ZP: Zone percentage
